# Discontinuation of Routine Distribution of Printed Copies of *MMWR Weekly*

**Published:** 2014-07-18

Effective August 1, 2014, subscribers who currently receive printed copies of *MMWR* at no cost directly from CDC each week will no longer receive them. This decision was reached after careful consideration of the costs of printing and mailing. Distribution of printed copies of *MMWR* serial publications (i.e., *Recommendations and Reports*, *Surveillance Summaries*, *Supplements*, and *Summaries of Notifiable Diseases*) was discontinued in 2011 (1).

All *MMWR* publications are available at no cost online at http://www.cdc.gov/mmwr. Readers desiring to have electronic copies of *MMWR* publications e-mailed to them automatically can subscribe online at http://www.cdc.gov/mmwr/mmwrsubscribe.html. Printed copies of the *MMWR Weekly* and serial publications are available by subscription from the Massachusetts Medical Society and from the U.S. Government Printing Office at http://www.cdc.gov/mmwr/order.html.
